# Supplementary material for: The development of laparoscopic skills using virtual reality simulations: A systematic review
Source: PLoS One. 2021 Jun 17;16(6):e0252609. doi: 10.1371/journal.pone.0252609 (PMC8211221; doi:10.1371/journal.pone.0252609)
Supplement: Attachment — Submitted filename: Response to Reviewers.docx [file pone.0252609.s002.docx]

Dear reviewers,

Thank you for inviting us to submit a revised draft of our manuscript.

We also appreciate the time and effort you and each of the reviewers have dedicated to providing insightful feedback on ways to strengthen our paper. Thus, it is with great pleasure that we resubmit our article for further consideration. We have incorporated changes that reflect the detailed suggestions you have graciously provided. We also hope that our edits and the responses we provide below satisfactorily address all of the issues and concerns you and the reviewers have noted.

**Reviewer #1: This systematic review article summarizes the results on the use of virtual reality simulator for the development of laparoscopic skills. A total of 1529 articles were collected and out of which 7 were selected after the exclusion criteria. The authors conclude that based on the seven articles, that virtual reality simulator doesn’t provide significant advantage over traditional training .**

**Training in laparoscopic surgery can vary from basic to advanced procedural skills. Moreover, virtual reality simulators are not uniform with some are designed to teach basic skills and some has both basic as well as advanced skills. The metrics used for assessing can also vary significantly between the simulators. Not all simulators have force feedback. Moreover, fidelity of these simulators is also very important (ie., whether they have undergone construct validity). These issues greatly limit the usefulness of metal analysis since the number of articles were going to be always very few.**

**The authors have summarized the results from the 7 papers but my concern is that the conclusion is severely limited by the factors mentioned above. Unlike the authors mentioned in the paper, there has been a review published on the virtual reality training for laparoscopic surgery (Alaker M, Wynn GR, Arulampalam T. Virtual reality training in laparoscopic surgery: A systematic review & meta-analysis. Int J Surg. 2016 May;29:85-94.). Also, the authors have cited the paper by Nagendran et al., which does a meta analysis of virtual reality training in laparoscopic surgery with focus on randomized clinical trials with primary and secondary outcomes but failed to elaborate on it. In view of the two review papers that has been published and the current review paper which by exclusion criteria doesn’t include papers beyond 2015, the real impact of the metal analysis is minimal. At the least, the authors should compare the conclusions from these two meta analysis to their conclusions and explain clearly any discrepancies.**

Thank you for pointing out this review from Alaker M *et al.* (2016). Indeed, we agree that there was a need to go more in depth in the existing literature. In order to address this issue, we have expanded the section of our introduction in which we discuss the standing of our review in relation to the ones done prior. Furthermore, as you suggested, other changes have been made in our discussion, considering the results of our review and how they compare to the previous ones. We believe that our paper has significantly improved with these adjustments, your feedback was much appreciated.

**The authors mention that in the article by Diesen et al., they didn’t report the VRS used in the study, but they did mention it in the discussion section (3rd paragraph). They have used both the Medical Education Technologies Inc. laparoscopic simulator as well as the Immersion virtual reality laparoscopic simulator. One would expect a thorough review of the paper especially for meta analysis. This needs to be fixed if the authors decided to revise the paper.**

You are right, we have updated this in our review and made a thorough revision of our paper for any other misplaced information.

**Reference 13 is incomplete and is also listed as part of reference 12.**

This has been fixed and now both references 12 and 13 are complete.

**Reviewer #2: I believe that this is an important piece. It is absolutely shocking that after 15 years of marketing and pushing for laparoscopic simulation the most we can find in the literature at this point to support its use is 7 papers. The number of poorly constructed C and D level publications in this space is simply incredible. The sections of the paper breaking down these shortcomings is a call-to-arms for those in this space to structure their research correctly and to power the studies appropriately. Thank you. There are a few grammatical issues in the paper that should be reviewed; a number of past/present tense inconsistencies as well as some simple capitalizations in the middle of sentences, etc.**

We agree with you and hope that our research can play its role in helping the scientific community. Additionally, our paper was sent for grammatical correction to a british translator.
