# Supplementary material for: The development of laparoscopic skills using virtual reality simulations: A systematic review
Source: PLoS One. 2021 Jun 17;16(6):e0252609. doi: 10.1371/journal.pone.0252609 (PMC8211221; doi:10.1371/journal.pone.0252609)
Supplement: Attachment — Submitted filename: Response to Reviewers 2.docx [file pone.0252609.s003.docx]

Dear reviewers,

Thank you for inviting us to submit a revised draft of our manuscript.

We also appreciate the time and effort you have dedicated to providing insightful feedback on ways to strengthen our paper. Thus, it is with great pleasure that we resubmit our article for further consideration. We have incorporated changes that reflect the detailed suggestions you have provided. We also hope that our edits and the responses we provide below satisfactorily address all of the issues and concerns you have previously noted.

**Reviewer #3: This systematic review article summarizes the results of using virtual reality simulators for developing laparoscopic skills from1529, of which, only 7 were selected. The authors concluded that virtual reality simulator has no significant edge over traditional training. I believe that this topic is important, and such review is needed.**

**Despite not including studies beyond 2015, the authors stated that no eligible studies were found. I belive this point should be highlighted in the abstract.**

**I also advise the authors to update their manuscript with the following references:**

**1- Gurusamy K, Aggarwal R, Palanivelu L, Davidson BR. Systematic review of randomized controlled trials on the effectiveness of virtual reality training for laparoscopic surgery. British Journal of Surgery. 2008 Sep;95(9):1088-97.**

**2- Jin C, Dai L, Wang T. The application of virtual reality in the training of laparoscopic surgery: a systematic review and meta-analysis. International Journal of Surgery. 2020 Dec 9.**

**Finally, I would also suggest that the paper be reviewed by a native English speaker to adjust for some grammatical errors.**

Indeed, we do believe the abstract should inform our readers about the lack of eligible studies beyond 2015, as this data is relevant and they could benefit from it beforehand. Hence we have incorporated your suggestion within the abstract limitations section.

Also, thank you for pointing out these two references, as they significantly improved our research and have been included within our text. Finally, our paper was once again revised by another native english-speaking translator and we hope all grammatical issues have now been corrected.

**Reviewer #1: The authors have addressed some of the issues that were noted in their initial submission. Specifically, added reference to a relevant meta-analysis paper by Alaket et al. With respect to new information that can be obtained from this meta analysis, compared to the other paper even though there is a clear four years difference, is very minimal. Even more, in this paper there is no meta-analysis in the end which is a big let down and results were more a qualitative summary.**

We have considered all your suggestions and really appreciate the consideration for our research, thank you for providing us with another significant point of view, which inspired us to seek improvements for elevating the quality of our research. We hope now that our paper meets your expectations with the newest changes and improvements, just as we expect that some of our decisions regarding other changes you proposed have been properly explained

**Discussion of cost of simulators is perfectly fine but over time, VR simulator can save cost for the hospital by reducing or eliminating the need for proctors, no need to replenish materials such has synthetic, cadaveric or animal tissues or organs etc. (advantages of VRS).**

Although the main goal of our review was not to analyse the cost-effectiveness of VRS, we believe that such considerations are of great importance and, as such, agree on the necessity to explore this subject. As you suggested, there was a need to expand our discussion on the matter of advantages of VRS and we, accordingly, addressed it. We now hope this segment of our review pleases and offers a more appropriate understanding of the question at hand.

**I think comparing to the existing meta-analysis, this review paper doesn't add more information and may be premature at this point for publication unless criteria of selection is changed to be able to perform an actual meta-analysis.**

Regarding the changes in eligibility criteria you suggested, we believe that, given the current stage of review, such changes would be unviable, as they would either demand a complete do over of the entire project or be included in such a late stage, as to compromise the impartiality of the paper. It should also be noted that unlike Alaker *et al.*, the other review you presented, we opted for a more restrictive eligibility criteria that explicitly required both pre and post-assessment for every study we included, in order to favor quality over quantity.

In the near future, we hope to once more submit other projects and studies to this Journal, making sure to take into account all suggestions you made early on and, certainly, your remarks will inspire us to continue seeking the betterment of any paper we may produce.
